# Multipathogen antimicrobial resistance in a Romanian tertiary-care hospital: a retrospective antibiogram analysis, January–July 2025

**DOI:** 10.25122/jml-2026-0074

**Published:** 2026-08

**Authors:** Cristiana Ana-Maria Olguța Penea, Violeta Melinte, Elena Crețu, Tiberiu Holban, Adelina Maria Radu, Cristina Maria Văcăroiu, Claudia Simona Cambrea, Valeriu Gheorghiță

**Affiliations:** 1 Prof. Dr. Agrippa Ionescu Clinical Emergency Hospital, Bucharest, Romania; 2 Carol Davila University of Medicine and Pharmacy, Bucharest, Romania; 3 Faculty of Medicine, Ovidius University of Constanța, Constanța, Romania

**Keywords:** antimicrobial resistance, multipathogen surveillance, *Klebsiella pneumoniae*, *Enterococcus faecium*, vancomycin-resistant enterococci, methicillin-resistant *Staphylococcus aureus*, cefiderocol, Romania, AMR, antimicrobial resistance, AmpC, AmpC-type β-lactamase, ESBL, extended-spectrum β-lactamase, EUCAST, European Committee on Antimicrobial Susceptibility Testing, GNB-Ent, Gram-negative bacilli – Enterobacterales, GNB-NF, non-fermenting Gram-negative bacilli, GPC, Gram-positive cocci, I, susceptible with increased exposure, ICU, intensive care unit, MDR, multidrug-resistant, MIC, minimum inhibitory concentration, MRS, methicillin-resistant staphylococci, MRSA, methicillin-resistant *Staphylococcus aureus*, MRSE, methicillin-resistant *Staphylococcus epidermidis*, R, resistant, S, susceptible, TMP-SMX, trimethoprim–sulfamethoxazole, VRE, vancomycin-resistant enterococci, XDR, extensively drug-resistant

## Abstract

Routine microbiology data can show not only isolated resistance events but also the wider hospital ecology in which antimicrobial resistance (AMR) emerges. We analyzed the complete January–July 2025 antibiogram export from a Romanian tertiary-care hospital, along with a targeted extended-testing file. The scope was deliberately broader than a single-organism carbapenem-resistant *Klebsiella pneumoniae* analysis. We performed a retrospective descriptive analysis using 3,270 antibiogram records from 3,012 patient identifiers. Organisms, sample types, resistance labels, and extended-test results were extracted from laboratory text fields. Organism occurrences within polymicrobial reports were counted separately to describe the full pathogen spectrum. The dataset contained 4,039 organism occurrences. Enterobacterales were the largest group, followed by Gram-positive cocci, non-fermenting Gram-negative bacilli, and yeasts. *Escherichia coli* and *K. pneumoniae* were the most frequent Enterobacterales, and clinically relevant resistant organisms were also identified among *Pseudomonas aeruginosa, Enterobacter cloacae* complex, *Staphylococcus aureus, Staphylococcus epidermidis, Enterococcus faecium* and *Candida* species. The extended-testing file contained 100 organism occurrences from 85 patient identifiers, mainly extensively drug-resistant Gram-negative isolates. Cefiderocol testing corresponded predominantly to *K. pneumoniae, P. aeruginosa* and *Acinetobacter baumannii* complex, whereas aztreonam/avibactam was concentrated mainly in extensively drug-resistant Enterobacterales, especially *K. pneumoniae*. The data support a multipathogen AMR interpretation: carbapenem-resistant Enterobacterales, non-fermenters, resistant Gram-positive cocci and yeasts coexist in the same hospital ecology. Empirical therapy, infection-control actions and stewardship decisions should be guided by syndrome, sample source, prior colonization and local species-level resistance patterns.

## INTRODUCTION

Antimicrobial resistance in hospitals is rarely confined to one bacterial species. In daily practice, the same clinical environment can contain carbapenem-resistant Enterobacterales, extensively drug-resistant non-fermenting Gram-negative bacilli, methicillin-resistant staphylococci, vancomycin-resistant enterococci and clinically important yeasts. These organisms differ in biology and treatment, but they share several practical drivers: antibiotic exposure, prolonged hospitalization, high-risk wards, invasive devices, recurrent cultures and repeated contact with healthcare systems [1–5].

*Klebsiella pneumoniae* remains one of the most important organisms because its natural cycle combines intestinal carriage, nosocomial transmission and carbapenemase-mediated resistance. However, focusing on Klebsiella alone can underestimate the diagnostic and therapeutic complexity seen in the microbiology laboratory. *Escherichia coli* contributes substantially to urinary and bloodstream infections; *Pseudomonas aeruginosa* and *Acinetobacter baumannii* complex are major problems in respiratory, intensive care unit (ICU) and device-associated infections; vancomycin-resistant enterococci (VRE), chiefly *Enterococcus faecium*, complicate severe nosocomial infections; *Staphylococcus aureus* and coagulase-negative staphylococci raise issues of methicillin resistance and device-associated disease; *Candida* species indicate fungal selection pressure in vulnerable hosts [6–11].

For this reason, the present analysis treats the dataset as a hospital-level antimicrobial resistance (AMR) ecosystem. It retains the *Klebsiella pneumoniae* data but places them alongside the other pathogens in the wider antibiogram export. This yields a more clinically useful message: the local resistance burden is multipathogen, and targeted testing such as cefiderocol or aztreonam/avibactam should be interpreted according to the organism and resistance mechanism it corresponds to.

The dataset can be read as a map of microbiological pressure within the hospital: common community-associated organisms, healthcare-associated resistant Gram-negative bacilli, device-related Gram-positive cocci and yeasts appear together, but they do not carry the same diagnostic or therapeutic implications. The analysis therefore emphasizes both frequency and clinical meaning, rather than simply listing organisms by abundance.

## MATERIAL AND METHODS

### Study design and data source

This was a retrospective laboratory-based observational analysis of routine microbiological antibiogram data collected between 1 January and 31 July 2025. The primary data source was the complete antibiogram export containing all routine antimicrobial susceptibility reports during the period. A second file containing targeted cefiderocol, aztreonam/avibactam and related salvage-agent testing was analyzed separately and then linked conceptually to the pathogens present in the complete dataset.

The analysis was designed to describe the pathogen spectrum, sample-source distribution, resistance phenotypes and advanced susceptibility testing across the broader microbiological dataset. Therefore, we used organism occurrences rather than unique patients for the main pathogen distribution tables (Supplementary Tables 1 and 2). This approach is appropriate when the objective is ecological surveillance of pathogens recovered by the laboratory.

Supplementary Tables

The unit of analysis was deliberately described as a laboratory observation rather than a confirmed infection episode. This distinction is important because urine, rectal screening, wound, respiratory, and blood-culture isolates have different clinical implications. A rectal isolate may indicate colonization and transmission risk, whereas a blood-culture isolate usually indicates invasive disease; both are relevant for antimicrobial-resistance surveillance, but they should not be interpreted in the same way.

### Organism extraction and harmonization

Pathogen names were extracted from the free-text antibiogram fields. Multiple organisms reported in the same microbiological record were separated and counted individually. Nomenclature was harmonized so that variants such as “*Klebsiella pneumoniae* ssp. pneumoniae” were grouped under *Klebsiella pneumoniae*. Resistance labels reported by the laboratory, including extensively drug-resistant (XDR), multidrug-resistant (MDR), extended-spectrum beta-lactamase (ESBL), AmpC beta-lactamase (AmpC), methicillin-resistant *Staphylococcus aureus* (MRSA), methicillin-resistant *Staphylococcus epidermidis* (MRSE), methicillin-resistant staphylococci (MRS) and vancomycin-resistant enterococci (VRE), were retained as phenotypic descriptors. All of these labels were those issued by the laboratory information system; they were retained as reported and were not independently reclassified.

### Sample-source categories

Microbiological tests were grouped into clinically interpretable sample-source categories: urine culture, rectal screening, respiratory specimen, blood culture, wound or soft tissue secretion, sterile fluid or collection, device-associated sample, screening/carriage and other samples. These categories allow the same pathogen to be interpreted in relation to likely clinical syndromes. For example, *Escherichia coli* was expected to be concentrated in urine cultures, whereas *Pseudomonas aeruginosa, Acinetobacter baumannii* complex and resistant *Klebsiella pneumoniae* are more relevant in respiratory, ICU, rectal screening or device-associated settings.

### Antimicrobial susceptibility and extended testing

Routine antimicrobial interpretations were extracted as susceptible, intermediate, or resistant where available and interpreted according to the EUCAST breakpoint tables, version 15.0 (2025) [12], which applied throughout the study period; no breakpoint version changed during the study. Data extraction and analysis were performed using Python (Python Software Foundation, Wilmington, DE, USA). The extended-testing file was used to evaluate high-priority agents, especially cefiderocol and aztreonam/avibactam. These tests were not treated as universal tests for all pathogens. They were interpreted as targeted investigations, mainly for highly resistant Gram-negative organisms where conventional options are limited. Tests for linezolid, vancomycin, teicoplanin and tigecycline in Gram-positive organisms were retained when they appeared in the same extended-testing file, but they were interpreted according to the organism involved.

For clarity, the article separates three levels of information: the complete pathogen ecology from the full export; clinically important resistance phenotypes by organism; and the smaller, targeted extended-testing subset. This avoids mixing routine surveillance counts with selected salvage-therapy tests.

## RESULTS

### Study overview and dataset structure

The complete export contained 3,270 antibiogram records and 4,039 organism occurrences. The difference between records and organism occurrences reflects polymicrobial reports: 703 records contained more than one organism. Figure 1 shows how the analytical dataset for the multipathogen analysis was derived.

**Figure 1:**
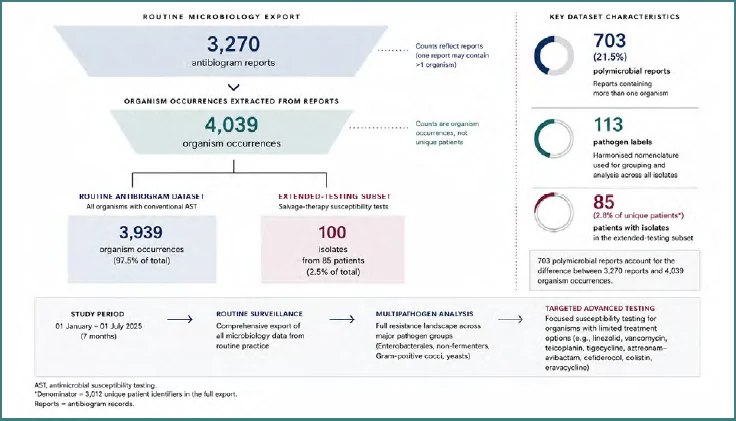
Study overview and dataset structure. Counts are organism occurrences unless otherwise specified; personal identifiers were used only for counting and were not reproduced.

### Overall microbiological structure of the dataset

Enterobacterales represented the dominant microbiological group. Gram-positive cocci, non-fermenting Gram-negative bacilli, fungi, and other bacteria were also identified. Figure 2 shows the broad microbiological group distribution in the complete antibiogram export. The broad distribution comprised Enterobacterales (46.2%), Gram-positive cocci (36.3%), non-fermenting Gram-negative bacilli (7.9%), fungi (7.3%) and other bacteria (2.4%).

**Figure 2:**
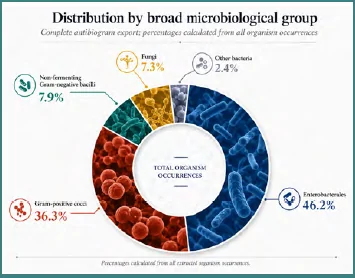
Broad microbiological group distribution in the complete antibiogram export. Percentages were calculated from all extracted organism occurrences.

### Main pathogens identified

The most frequent organisms were *Escherichia coli* and *Klebsiella pneumoniae*, followed by *Staphylococcus aureus, Pseudomonas aeruginosa, Enterococcus faecalis, Staphylococcus epidermidis* and *Enterococcus faecium*. Figure 3 presents the most frequent pathogens recovered during the study period. *Escherichia coli* accounted for 24.0% of all organism occurrences and *Klebsiella pneumoniae* for 11.1%. The leading Gram-positive organisms were *Staphylococcus aureus* (9.5%), *Enterococcus faecalis* (5.9%), *Staphylococcus epidermidis* (5.7%) and *Enterococcus faecium* (5.1%), while *Pseudomonas aeruginosa* accounted for 5.6% and *Candida albicans* for 3.3%.

**Figure 3:**
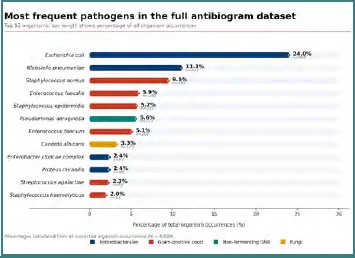
Most frequent pathogens recovered during the study period. Percentages and occurrence counts were calculated from all extracted organism occurrences.

### Resistance phenotypes across pathogens

Resistance labels included XDR, MDR/ESBL-related phenotypes, methicillin resistance, and VRE. Figure 4 shows the resistance phenotype matrix for Gram-positive cocci. Methicillin resistance was identified in 104/382 *Staphylococcus aureus* isolates (27.2%), 158/232 *Staphylococcus epidermidis* isolates (68.1%), 65/81 *Staphylococcus haemolyticus* isolates (80.2%) and 18/30 other coagulase-negative staphylococci (60.0%). Vancomycin resistance was concentrated in *Enterococcus faecium* (156/207, 75.4%) and was uncommon in *Enterococcus faecalis* (6/240, 2.5%). Among Enterobacterales, 39.5% of *Klebsiella pneumoniae* isolates were resistant to meropenem and 41.8% to ertapenem, whereas carbapenem resistance in *Escherichia coli* was rare (0.9% to meropenem, 1.7% to ertapenem).

**Figure 4:**
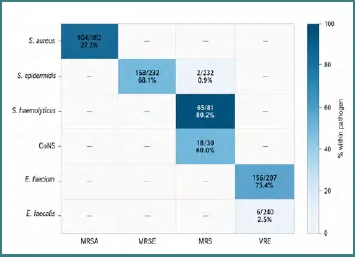
Resistance phenotype matrix for Gram-positive cocci. Cell labels show the number of isolates with the phenotype over the number of isolates of that species, with the percentage below. Color intensity reflects the percentage within each pathogen. A dash indicates that the phenotype does not apply to that organism. Abbreviations: CoNS, coagulase-negative staphylococci; MRS, methicillin-resistant staphylococci; MRSA, methicillin-resistant Staphylococcus aureus; MRSE, methicillin-resistant Staphylococcus epidermidis; VRE, vancomycin-resistant enterococci.

### Sample-source distribution

Sample-source distribution by microbiological class is presented in Figure 5. Counts are organism occurrences, not unique patients. Urine culture was the dominant source for Enterobacterales (917/1718, 53.4%). Wound or soft tissue samples were the dominant source for non-fermenting Gram-negative bacilli (97/290, 33.4%) and Gram-positive cocci (608/1235, 49.2%), whereas respiratory specimens were the dominant source for yeasts/fungi (42/135, 31.1%).

**Figure 5:**
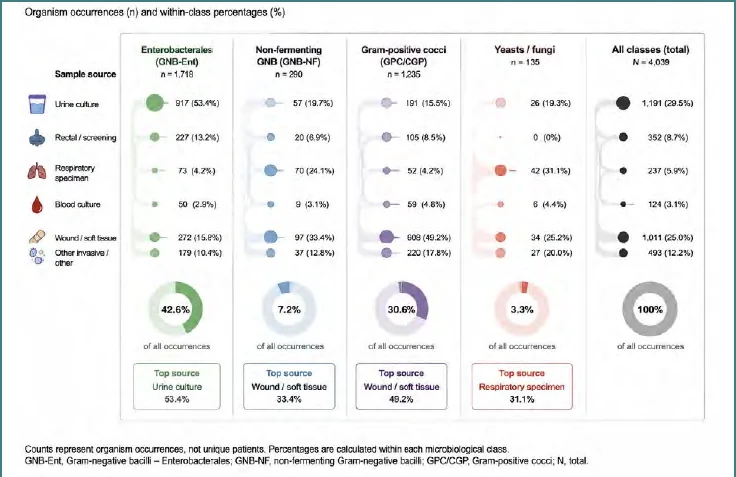
Sample–source distribution by microbiological class. Counts represent organism occurrences, not unique patients. Percentages are calculated within each microbiological class. The zero-occurrence sterile fluid/collection row was omitted from the display.

### Correspondence of extended tests with pathogens

The extended-testing file contained 100 organism occurrences from 85 patient identifiers. Figure 6 shows the pathogen correspondence between the complete antibiogram dataset and the extended-testing subset. The extended-testing subset was dominated by Enterobacterales (81/100), mainly *Klebsiella pneumoniae* (73 occurrences). Non-fermenting Gram-negative bacilli accounted for 13/100 occurrences and Gram-positive cocci for 6/100 occurrences.

**Figure 6:**
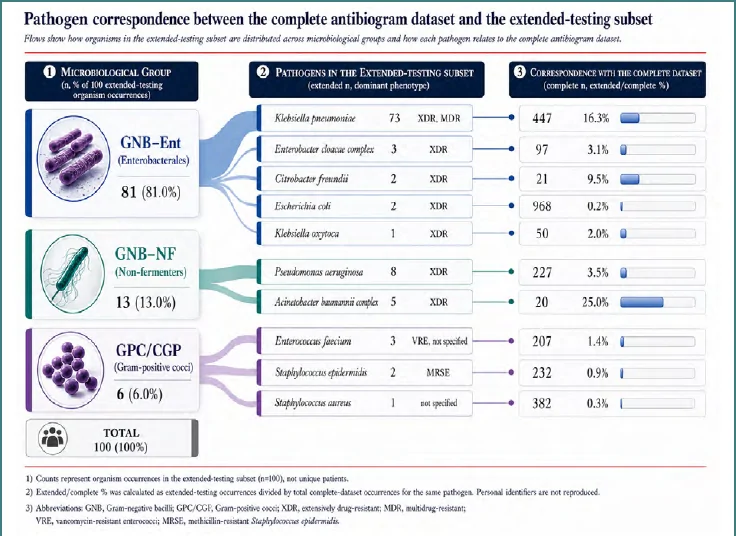
Pathogen correspondence between the complete antibiogram dataset and the extended-testing subset. Counts represent organism occurrences in the extended-testing subset, not unique patients. Extended/complete percentages were calculated using the complete-dataset denominator for each pathogen.

### Results of cefiderocol, aztreonam/avibactam and related targeted testing

Figure 7 summarizes the targeted antimicrobial testing results by pathogen-agent pair. Bubble labels show susceptible/tested isolates; bubble size reflects the number of tested isolates, and color reflects the susceptible proportion. Only the principal advanced and salvage agents are shown; rare comparator agents are provided in Supplementary Table S2.

**Figure 7:**
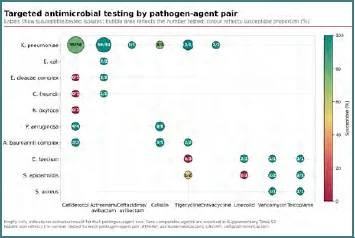
Targeted antimicrobial testing by pathogen-agent pair. Bubble labels show susceptible/tested isolates. Bubble size reflects the number of tested isolates for each pathogen-agent pair, and color reflects the susceptible proportion. Empty cells indicate that no extracted result was available for that pathogen-agent combination.

Among Gram-negative targeted tests, *Klebsiella pneumoniae* accounted for most cefiderocol and aztreonam/avibactam results. Cefiderocol susceptibility was 39/50 for *Klebsiella pneumoniae* and 4/4 for *Pseudomonas aeruginosa*, while aztreonam/avibactam susceptibility was 39/39 for *Klebsiella pneumoniae*. Colistin results were 3/4 for *Klebsiella pneumoniae*, 8/8 for *Pseudomonas aeruginosa*, and 5/5 for *Acinetobacter baumannii* complex. Gram-positive targeted agents were represented by linezolid, vancomycin, and teicoplanin results.

## DISCUSSION

### The common theme: a hospital AMR ecosystem

The analysis was based on organism occurrences rather than unique patients, so polymicrobial reports contribute more than one observation. This is clinically important because polymicrobial results often occur in urine, wound, respiratory, or device-associated samples and can change antimicrobial decisions.

Considering all organisms together broadens the picture. The resistance problem is not limited to carbapenem-resistant *Klebsiella pneumoniae*; the data show simultaneous pressures from ESBL-producing Enterobacterales, non-fermenting Gram-negative bacilli with difficult-to-treat profiles, methicillin-resistant staphylococci, vancomycin-resistant enterococci and clinically relevant *Candida* species. Such coexistence is typical of tertiary-care settings where antibiotic exposure, severe underlying disease and invasive procedures overlap.

Enterobacterales represented the dominant microbiological group, but the dataset was not limited to Enterobacterales. Gram-positive cocci, non-fermenting Gram-negative bacilli, and fungi were also frequent, supporting treatment as a multipathogen rather than a single-organism resistance problem.

The rank order is clinically plausible. *Escherichia coli* predominates because urinary isolates are common in routine microbiology, while *Klebsiella pneumoniae* remains highly visible because it is more strongly linked to hospital acquisition, colonization pressure, and carbapenemase testing. *Staphylococcus aureus* and coagulase-negative staphylococci reflect skin, wound, catheter and device-related sampling, while *Enterococcus* and *Candida* species point toward complex patients with previous antibiotic exposure, urinary instrumentation, abdominal pathology or prolonged admission.

The common element linking these organisms is not taxonomic similarity but clinical ecology. The dataset describes a hospital environment in which antibiotic exposure, carriage pressure, invasive care and repeated sampling create opportunities for resistant organisms to appear across multiple biological groups. This is why the most clinically coherent theme is a multipathogen antimicrobial-resistance landscape rather than a *Klebsiella*-only story.

In this sense, the dataset illustrates the continuum between colonization and infection. Resistant Enterobacterales may first be detected by screening, and later found in urine, respiratory samples, or blood cultures. Coagulase-negative staphylococci may represent contamination in some contexts, but in the presence of prosthetic material or intravascular devices they become clinically meaningful. *Candida* recovered from non-sterile sites may represent colonization, while *Candida* in blood culture or deep sterile samples changes management completely. The medical interpretation depends on the organism, source, and patient context.

From a bedside perspective, this matters because the same patient profile may be vulnerable to different pathogens at different moments. A patient with urinary symptoms may grow *Escherichia coli* or *Klebsiella pneumoniae*; a ventilated or ICU patient may grow *Pseudomonas aeruginosa, Acinetobacter baumannii* complex or XDR *Klebsiella pneumoniae*; a patient with a catheter or bloodstream infection may require consideration of *Staphylococcus epidermidis* MRSE, *Staphylococcus aureus* MRSA, *Enterococcus faecium* VRE or *Candida* species. These organisms are not interchangeable, but they are linked by the same hospital pressures.

### Klebsiella pneumoniae as an anchor, not the only focus

*Klebsiella pneumoniae* remains an anchor pathogen because it is frequent, clinically important, and strongly represented among XDR and advanced-testing results. The species also has high infection-control relevance because intestinal carriage can precede infection and because carbapenemase-producing strains can disseminate in healthcare settings. However, the broader export shows that *Klebsiella pneumoniae* should be interpreted within a network of organisms. A stewardship strategy aimed only at *Klebsiella* would miss ESBL *Escherichia coli*, XDR *Pseudomonas aeruginosa*, VRE *Enterococcus faecium*, MRSA/MRSE staphylococci and *Candida auris* [7,9–11].

*Klebsiella pneumoniae* is best understood as one component of the larger ecology. It is the clearest marker of carbapenemase-driven Enterobacterales pressure, particularly where XDR labels and aztreonam/avibactam or cefiderocol testing are present. It should not, however, obscure the contribution of *Escherichia coli* to the urinary burden, of *Pseudomonas* and *Acinetobacter* to difficult respiratory or ICU infections, of *Enterococcus* to device- and abdominal-associated disease, and of *Candida* to the fungal burden of complex hospitalized patients.

### Enterobacterales beyond Klebsiella

*Escherichia coli* was the most frequent organism in the dataset. Its clinical weight is different from *Klebsiella pneumoniae*: it is heavily represented in urinary infections and community-associated or healthcare-associated urinary syndromes, but resistant ESBL phenotypes can transform a usually manageable infection into one requiring broader therapy. *Enterobacter cloacae* complex, *Serratia marcescens, Proteus mirabilis, Morganella morganii* and *Citrobacter freundii* are less frequent but clinically relevant because intrinsic resistance mechanisms, AmpC production or acquired β-lactamases may narrow treatment options.

The Enterobacterales group therefore forms a spectrum: common urinary pathogens at one end, highly resistant carbapenemase-producing organisms at the other, and several intermediate organisms with inducible or acquired resistance mechanisms.

### Resistance phenotypes and sample-source interpretation

Resistance labels were not distributed randomly. XDR phenotypes were concentrated mainly among *Klebsiella pneumoniae* and other difficult-to-treat Gram-negative bacilli. ESBL and MDR-associated ESBL phenotypes were most relevant for Enterobacterales. MRSA described resistant *Staphylococcus aureus*, whereas MRSE and MRS labels were linked to coagulase-negative staphylococci. VRE was especially relevant for *Enterococcus faecium*.

From a medical perspective, the phenotype label should be interpreted together with the organism. ESBL in *Escherichia coli* usually raises questions about urinary-source treatment, oral step-down options, and prior antibiotic exposure. XDR *Klebsiella pneumoniae* or XDR *Pseudomonas aeruginosa* raises different concerns: limited empirical coverage, the need for rapid escalation, and a possible requirement for extended testing. MRSA, MRSE, and VRE represent another therapeutic axis, where glycopeptides, linezolid, daptomycin, or other Gram-positive-active agents may become relevant depending on infection site and severity.

This pattern has practical consequences. In an unstable patient, the likely pathogen cannot be inferred from resistance terminology alone. A patient with VRE requires a completely different therapeutic pathway from a patient with XDR *Klebsiella pneumoniae* or XDR *Pseudomonas aeruginosa*, even though all three are markers of hospital-associated resistance pressure.

Sample source helps translate laboratory ecology into bedside meaning. Urine cultures dominated the recovery of *Escherichia coli* and a substantial fraction of *Klebsiella pneumoniae*. Rectal screening was particularly relevant for resistant Enterobacterales carriage. Respiratory specimens and ICU-related contexts were more important for *Pseudomonas aeruginosa, Acinetobacter baumannii* complex, and resistant Gram-negative organisms. Blood cultures, although less frequent than urine or screening cultures, are clinically high-impact because they represent invasive disease rather than colonization.

The sample-source distribution also helps avoid overinterpretation. A high number of urine isolates does not automatically mean severe infection, whereas fewer bloodstream isolates may carry disproportionate clinical importance. Respiratory isolates, especially in ventilated or ICU patients, require correlation with radiology, inflammatory markers and clinical status. Rectal screening isolates are valuable epidemiologically because they identify reservoirs for subsequent infection and for cross-transmission, even when the patient is not infected at the moment of sampling.

### Non-fermenting Gram-negative bacilli

*Pseudomonas aeruginosa* and *Acinetobacter baumannii* complex require separate clinical interpretation. Their resistance is not simply an extension of ESBL or *Klebsiella* carbapenemase biology. These organisms have permeability barriers, efflux mechanisms and complex intrinsic resistance profiles. In practice, they are especially important in ICU, respiratory, chronic wound and device-associated settings. The presence of cefiderocol and colistin testing for these organisms in the extended file is consistent with difficult-to-treat non-fermenter management [13–16].

For these pathogens, intrinsic resistance is as important as acquired resistance. *Pseudomonas aeruginosa* may remain susceptible to selected antipseudomonal beta-lactams in some cases, but MDR or XDR phenotypes narrow treatment rapidly. *Acinetobacter baumannii* complex is particularly problematic because resistance often involves multiple mechanisms at once, making routine susceptibility interpretation clinically decisive. Their presence supports the need to separate non-fermenters from Enterobacterales in both tables and discussion.

*Stenotrophomonas maltophilia* also deserves mention. It is intrinsically resistant to many broad-spectrum β-lactams, including carbapenems, and should not be treated as a standard *Pseudomonas*-like isolate. Its presence in the dataset supports organism-specific interpretation rather than broad Gram-negative generalizations.

### Gram-positive resistance and device-associated disease

The Gram-positive component is dominated by *Staphylococcus aureus*, coagulase-negative staphylococci and enterococci. MRSA remains important because it changes empirical coverage decisions for skin, soft tissue, respiratory and bloodstream syndromes. MRSE and methicillin-resistant coagulase-negative staphylococci may represent contamination in some blood cultures, but they are clinically important when associated with catheters, prosthetic material or repeated positive cultures, and should not be dismissed as contaminants on the basis of species alone [3,4,14].

This group also reinforces the importance of clinical adjudication. *Staphylococcus aureus* should rarely be dismissed when recovered from blood or deep samples, whereas coagulase-negative staphylococci require correlation with the number of positive cultures, device presence and inflammatory signs. Enterococci are especially relevant in urinary, intra-abdominal and bloodstream contexts, and VRE status changes both infection-control measures and treatment choices.

*Enterococcus faecium* VRE is one of the clearest hospital-associated resistance signals in the dataset. Compared with *Enterococcus faecalis, E. faecium* is more likely to be resistant to ampicillin and vancomycin, leaving linezolid or other targeted agents as clinically important options. The extended-testing file correctly contains linezolid/glycopeptide-type testing for Gram-positive organisms, which should be kept separate from cefiderocol or aztreonam/avibactam interpretation.

### Candida species and the fungal dimension

The presence of *Candida albicans, Candida glabrata* and *Candida auris* confirms that the dataset also captures fungal selection and colonization/infection patterns in vulnerable patients.

*Candida* species add another layer to the hospital ecology. *Candida albicans* was the most frequent yeast, but *Candida glabrata* and *Candida auris* are clinically important because they may be associated with antifungal resistance, outbreaks, colonization pressure, and complex infection-control requirements. In a multipathogen AMR article, *Candida* is not a side note: it signals that antimicrobial pressure includes antibacterial and antifungal selection in vulnerable patients [4,17,18].

The fungal findings are important because they often mark a different type of vulnerability: broad-spectrum antibiotic exposure, central venous catheters, parenteral nutrition, abdominal surgery, ICU admission or immunosuppression. Including *Candida* strengthens the multipathogen concept, because antimicrobial pressure does not select only bacteria; it can also promote fungal overgrowth and increase invasive candidiasis risk in selected patients.

### Clinical use of extended testing

The extended-testing file represents a selected subset rather than the whole microbiology population. Its results correspond mainly to highly resistant isolates, particularly XDR *Klebsiella pneumoniae* and other difficult-to-treat Gram-negative organisms. This explains why cefiderocol and aztreonam/avibactam appear in this file: they are generally requested when routine therapeutic options are limited, or when a carbapenemase or non-fermenter resistance pattern requires further clarification.

The correspondence is clinically meaningful. Aztreonam/avibactam results were concentrated in XDR Enterobacterales, especially *Klebsiella pneumoniae*, where metallo-β-lactamase or mixed carbapenemase contexts may make aztreonam-based combinations relevant. Cefiderocol was tested across *Klebsiella pneumoniae, Pseudomonas aeruginosa, Acinetobacter baumannii* complex and selected Enterobacterales. Colistin and tigecycline appeared as comparator or salvage agents, but interpretation must account for toxicity, infection site and pharmacokinetic limitations.

Therefore, the extended-testing table should be read as a selected high-risk subset. It does not estimate susceptibility for the entire hospital population; it describes the isolates for which routine results or resistance phenotype created a specific therapeutic question. This is why the same organism may appear in the full pathogen table and again in the extended-testing correspondence table, but with different denominators and different clinical meaning.

Gram-positive results in this file should not be interpreted through the same mechanism; linezolid, vancomycin, and teicoplanin correspond to *Enterococcus* or *Staphylococcus* isolates, not to Gram-negative carbapenemase pathways.

These results must be interpreted cautiously, because targeted testing is enriched for resistant isolates. A susceptible result in this subset is clinically reassuring but should still be read in relation to infection site, inoculum, renal function, source control and available alternatives. A resistant result is equally important, because it limits salvage options and may indicate complex resistance mechanisms, particularly where carbapenemase production, porin loss or efflux are suspected.

Cefiderocol and aztreonam/avibactam are targeted tools rather than general-purpose agents for this population. Cefiderocol is relevant for selected carbapenem-resistant Gram-negative organisms, including some non-fermenters. Aztreonam/avibactam is most logically applied in resistant Enterobacterales where β-lactamase combinations make usual β-lactams unreliable. Colistin, tigecycline, and eravacycline provide additional options, but their clinical use depends on infection site, severity, source control, pharmacokinetics, and toxicity [12–16,19–22].

Aztreonam/avibactam is particularly relevant when metallo-beta-lactamase activity is suspected, because aztreonam is stable to metallo-beta-lactamases while avibactam inhibits many co-produced serine beta-lactamases. Cefiderocol has a different rationale, using siderophore-mediated entry to overcome some permeability barriers in Gram-negative organisms. These mechanisms explain why the two tests belong in a targeted therapeutic section, not in the general routine antibiogram table [19–22].

Advanced tests correspond to specific organism–mechanism combinations and are best reported as a targeted subgroup rather than merged with routine susceptibility percentages.

### Clinical implications

- Empirical treatment should be guided by syndrome and sample source: urinary, bloodstream, respiratory, wound/device and screening samples have different pathogen probabilities.
- *Klebsiella pneumoniae* remains a key AMR indicator, but the local ecology also includes ESBL *Escherichia coli*, XDR *Pseudomonas aeruginosa*, resistant *Acinetobacter*, MRSA/MRSE, VRE and *Candida* species.
- Rectal screening and prior resistant isolates are clinically useful because colonization may precede infection, especially for resistant Enterobacterales.
- Extended testing should be requested and interpreted selectively. Cefiderocol and aztreonam/avibactam results are most informative when linked to a specific pathogen and resistance phenotype.
- Stewardship interventions should not focus only on carbapenems; they should also consider glycopeptides, linezolid, fluoroquinolones, TMP-SMX, antifungals and last-line Gram-negative agents.
- Infection control should treat the dataset as evidence of a shared hospital resistance ecosystem, including Gram-negative, Gram-positive and fungal threats.

### Limitations

This analysis is based on laboratory exports and therefore describes microbiological observations rather than adjudicated clinical infections. Some isolates may represent colonization, screening positivity or contamination, particularly for coagulase-negative staphylococci and *Candida* in non-sterile sites. Organism occurrences were counted separately in polymicrobial samples; this is appropriate for ecological surveillance but should not be interpreted as patient-level incidence. The extended-testing file is selected by clinical or laboratory indication and therefore cannot be used to estimate population-level susceptibility to cefiderocol or aztreonam/avibactam. Finally, resistance mechanisms were inferred from reported phenotypes unless molecular data were explicitly present in the laboratory result.

Another limitation is that resistance labels and susceptibility results depend on what was tested in routine practice. Some drugs were tested only in selected isolates, and extended tests were requested for clinically or microbiologically difficult cases. As a result, percentages from the targeted subset should not be generalized to all isolates. The strength of the dataset is breadth and real-world relevance, while the limitation is the absence of full clinical adjudication for every organism occurrence.

## CONCLUSION

The study supports a multipathogen interpretation of antimicrobial resistance in the examined tertiary-care hospital, with resistant Gram-negative bacilli, Gram-positive cocci and yeasts contributing to the local microbiological landscape. The principal rates were 39.5% meropenem resistance in *Klebsiella pneumoniae*, 75.4% vancomycin resistance in *Enterococcus faecium*, and 27.2% methicillin resistance in Staphylococcus aureus, 68.1% in Staphylococcus epidermidis, and 80.2% in *Staphylococcus haemolyticus. Klebsiella pneumoniae* remains an important resistance marker, but the dataset also highlights relevant contributions from *Escherichia coli, Pseudomonas aeruginosa, Acinetobacter baumannii* complex, *Staphylococcus* spp., *Enterococcus* spp. and *Candida* spp. Routine antibiogram data should be interpreted alongside pathogen identity, sample source, and clinical context. Extended testing should be considered a targeted layer for selected resistant isolates rather than a population-wide susceptibility estimate. Local surveillance remains important for guiding empirical therapy, antimicrobial stewardship and infection-control priorities.

## Data Availability

The anonymized aggregate data supporting this analysis are available from the corresponding author upon reasonable request and subject to institutional governance requirements.

## Personal thanks

This work was developed within the academic framework of the first author’s doctoral research. The authors thank the microbiology laboratory team for data support and routine diagnostic work.
