# Supplementary material for: Multipathogen antimicrobial resistance in a Romanian tertiary-care hospital: a retrospective antibiogram analysis, January–July 2025
Source: J Med Life. 2026 Aug;19(8):627–35. doi: 10.25122/jml-2026-0074 (PMC13674048; doi:10.25122/jml-2026-0074)
Supplement: Supplementary file 1 [file JMedLife-19-627-s001.pdf]

Supplementary Tables

Supplementary tables provide the detailed routine susceptibility and extended-testing data supporting the main text figures and tables.

Supplementary Table 1. Routine antimicrobial susceptibility of the principal pathogens recovered during the study period

Antimicrobial	*n* tested	S (%)	I (%)	R (%)
Escherichia coli				
Meropenem	964	99.1	0.0	0.9
Ertapenem	920	98.3	0.0	1.7
Ceftazidime	954	81.9	3.7	14.5
Cefepime	115	45.2	12.2	42.6
Ceftolozane/tazobactam	106	91.5	0.0	8.5
Piperacillin/tazobactam	455	84.2	0.0	15.8
Klebsiella pneumoniae				
Meropenem	446	58.5	2.0	39.5
Ertapenem	423	58.2	0.0	41.8
Ceftazidime	444	37.6	1.8	60.6
Cefepime	257	8.2	2.7	89.1
Ceftolozane/tazobactam	254	24.4	0.0	75.6
Piperacillin/tazobactam	377	37.9	0.0	62.1
Enterobacter cloacae complex				
Meropenem	97	95.9	0.0	4.1
Ertapenem	71	78.9	0.0	21.1
Ceftazidime	97	59.8	2.1	38.1
Cefepime	49	61.2	12.2	26.5
Ceftolozane/tazobactam	49	65.3	0.0	34.7
Piperacillin/tazobactam	91	58.2	0.0	41.8
Pseudomonas aeruginosa				
Meropenem	225	71.1	8.0	20.9
Ceftazidime	221	0.9	74.7	24.4
Cefepime	217	0.9	77.4	21.7
Ceftolozane/tazobactam	213	83.1	0.0	16.9
Piperacillin/tazobactam	222	0.9	68.5	30.6
Amikacin	195	98.5	0.0	1.5
Acinetobacter baumannii complex				
Meropenem	20	40.0	0.0	60.0
Amikacin	20	40.0	0.0	60.0
Gentamicin	20	40.0	0.0	60.0
Ciprofloxacin	20	0.0	40.0	60.0
Levofloxacin	15	26.7	6.7	66.7
Colistin	16	93.8	0.0	6.2
Stenotrophomonas maltophilia				
Trimethoprim–sulfamethoxazole	35	97.1	0.0	2.9
Levofloxacin	62	91.9	3.2	4.8
Staphylococcus aureus				
Gentamicin	382	96.9	0.0	3.1
Ciprofloxacin	17	0.0	100.0	0.0
Levofloxacin	365	1.9	90.1	7.9

© 2026 by the authors. This article is an open access article distributed under the terms and conditions of the Creative Commons Attribution (CC BY 4.0) license.

Supplementary Table 1. Continued. Routine antimicrobial susceptibility of the principal pathogens recovered during the study period

Antimicrobial	*n* tested	S (%)	I (%)	R (%)
Trimethoprim–sulfamethoxazole	382	97.4	0.3	2.4
Tigecycline	381	99.7	0.0	0.3
Vancomycin	381	100.0	0.0	0.0
Staphylococcus epidermidis				
Gentamicin	232	66.8	0.0	33.2
Ciprofloxacin	53	1.9	73.6	24.5
Levofloxacin	183	0.0	55.2	44.8
Trimethoprim–sulfamethoxazole	231	74.0	0.0	26.0
Tigecycline	231	99.1	0.0	0.9
Vancomycin	230	100.0	0.0	0.0
Enterococcus faecalis				
Ciprofloxacin	239	78.7	0.0	21.3
Levofloxacin	181	74.6	0.6	24.9
Vancomycin	237	97.5	0.0	2.5
Teicoplanin	236	97.5	0.0	2.5
Linezolid	238	100.0	0.0	0.0
Tigecycline	234	99.1	0.0	0.9
Enterococcus faecium				
Ciprofloxacin	205	8.3	0.0	91.7
Levofloxacin	197	6.6	0.0	93.4
Vancomycin	204	21.6	0.0	78.4
Teicoplanin	205	22.0	0.0	78.0
Linezolid	206	98.5	0.0	1.5
Tigecycline	202	92.6	0.0	7.4

I, susceptible with increased exposure; R, resistant; S, susceptible. Isolates are organism occurrences, not unique patients. Rows are shown only where at least 10 isolate–antimicrobial results were available; percentages are calculated among isolates tested for each pathogen–antimicrobial pair, so denominators differ between agents. Results were interpreted according to EUCAST breakpoint tables, version 15.0 (2025), which applied throughout the study period.

© 2026 by the authors. This article is an open access article distributed under the terms and conditions of the Creative Commons Attribution (CC BY 4.0) license.

Supplementary Table 2. Extended and salvage-agent susceptibility testing, by pathogen and antimicrobial.

Antimicrobial	*n* tested	S	I	R	Observed MIC values (mg/L)
Klebsiella pneumoniae					
Aztreonam/avibactam	39	39	0	0	<0.016, 0.094, 0.125, 0.19, 0.25, 0.38, 0.5, 0.75
Cefiderocol	50	39	0	11	0.125, 0.25, 0.5, 1, 2, 4, 8, 16
Ceftazidime/avibactam	1	1	0	0	≤1/4
Colistin	4	3	0	1	≤1, >8
Eravacycline	2	2	0	0	0.36, 0.38
Tigecycline	13	13	0	0	≤0.25, 0.5, 1
Escherichia coli					
Aztreonam/avibactam	2	2	0	0	1.5
Enterobacter cloacae complex					
Aztreonam/avibactam	2	2	0	0	0.032, 0.064
Cefiderocol	1	0	0	1	8
Citrobacter freundii					
Aztreonam/avibactam	1	1	0	0	0.023
Cefiderocol	1	0	0	1	4
Klebsiella oxytoca					
Cefiderocol	1	0	0	1	4
Pseudomonas aeruginosa					
Cefiderocol	4	4	0	0	1, 2
Colistin	8	8	0	0	<1, ≤1, 2, 4
Acinetobacter baumannii complex					
Cefiderocol	3	3	0	0	0.125, 0.25, 0.5
Colistin	5	5	0	0	≤1
Tigecycline	2	2	0	0	≤0.25, 0.5
Enterococcus faecium					
Linezolid	3	3	0	0	2
Teicoplanin	1	1	0	0	≤1
Vancomycin	1	1	0	0	≤1
Tigecycline	3	0	0	3	≥0.5, >0.5
Staphylococcus epidermidis					
Linezolid	2	0	0	2	>4, >8
Teicoplanin	2	2	0	0	≤1, 2
Vancomycin	2	2	0	0	≤1, 2
Tigecycline	2	1	0	1	0.25, 1
Staphylococcus aureus					
Teicoplanin	1	1	0	0	2
Vancomycin	1	1	0	0	2

I, susceptible with increased exposure; MIC, minimum inhibitory concentration; R, resistant; S, susceptible. Counts are organism occurrences, not unique patients. Denominators are the number of isolates tested for each pathogen–agent pair within the extended-testing file (100 organism occurrences from 85 patient identifiers) and are not the pathogen totals for the hospital export; this subset is selected by clinical or laboratory indication and is enriched for resistant isolates, so proportions must not be generalised to all isolates. Results were interpreted according to EUCAST breakpoint tables, version 15.0 (2025).

© 2026 by the authors. This article is an open access article distributed under the terms and conditions of the Creative Commons Attribution (CC BY 4.0) license.
